# From Benign to Lethal: A Case of Aggressive Choriocarcinoma With Systemic Metastases

**DOI:** 10.7759/cureus.105772

**Published:** 2026-03-24

**Authors:** Esperance M Madera, Parag Gajurel, Domonick K Gordon, Musa Ayyad, Jeffrey Lefko, Prathyusha Pinnamaneni, Morgan Moore, Karen Orenshteyn, Gautam Valecha, Manubolu Devanand, Avi Saini

**Affiliations:** 1 Internal Medicine, Mount Sinai Hospital, Chicago, USA; 2 Hematology and Oncology, Mount Sinai Hospital, Chicago, USA; 3 Internal Medicine, Scarborough General Hospital, Scarborough, TTO; 4 Internal Medicine, Ross University School of Medicine, Bridgetown, BRB

**Keywords:** choriocarcinoma, cyclophosphamide, dactinomycin, etoposide, metastasis, methotrexate, serum β-hcg, vincristine

## Abstract

Choriocarcinoma is a rare and aggressive gestational trophoblastic neoplasm that often arises after a molar pregnancy or miscarriage and is characterized by rapid metastatic potential. Diagnosis is typically based on clinical presentation, history of antecedent pregnancy, markedly elevated β-hCG levels, and supportive imaging and histopathologic findings. Early recognition and prompt initiation of multi-agent chemotherapy are crucial, as they markedly improve survival, with remission achievable even in advanced disease. We report the case of a 24-year-old woman who presented with persistent vaginal bleeding, severe pelvic pain, and progressive dyspnea one month after a molar pregnancy treated with dilation and curettage. Examination revealed pallor and mild abdominal tenderness. Laboratory studies showed a β-hCG level >250,000 mIU/mL (reference range: 0-5 mIU/mL) and profound anemia with a hemoglobin of 2 g/dL (reference range: 12-15.3 g/dL). Contrast-enhanced abdominal and pelvic computed tomography demonstrated metastatic lesions in the liver and gastrointestinal tract, while chest CT revealed multiple pulmonary nodules consistent with metastases. The patient was promptly initiated on multi-agent chemotherapy, resulting in normalization of β-hCG levels after several treatment cycles. This case highlights the importance of vigilant follow-up in patients with molar pregnancies and persistently elevated β-hCG, as timely diagnosis and treatment can lead to curative outcomes despite widespread metastasis.

## Introduction

Choriocarcinoma is a rare and highly aggressive form of gestational trophoblastic neoplasia (GTN), which most commonly arises following a molar pregnancy, miscarriage, ectopic pregnancy, or term gestation [1,2]. It is characterized by malignant proliferation of cytotrophoblastic and syncytiotrophoblastic cells without chorionic villi and demonstrates an early propensity for hematogenous dissemination, particularly to the lungs, liver, and brain [2,3].

Clinically, patients often present with abnormal uterine bleeding and markedly elevated serum β-human chorionic gonadotropin (β-hCG) levels that are disproportionate to gestational status [3,4]. Diagnosis is primarily clinical and biochemical, supported by imaging to evaluate metastatic spread. Established diagnostic algorithms incorporate serial β-hCG measurements, pelvic imaging, and systemic evaluation for distant metastases [4,5]. Histopathologic confirmation may be obtained when tissue is available, although treatment should not be delayed in the presence of characteristic clinical and biochemical findings.

Prognosis and treatment planning are guided by the International Federation of Gynecology and Obstetrics (FIGO) staging and scoring system, which stratifies patients into low- and high-risk categories based on factors such as antecedent pregnancy type, interval since pregnancy, pretreatment β-hCG levels, tumor size, and sites of metastasis [5,6]. This risk stratification system has significant prognostic utility and directs the selection of single-agent versus multi-agent chemotherapy regimens.

Despite its aggressive nature, choriocarcinoma is highly chemosensitive. With appropriate risk-adapted therapy, survival rates exceed 90% in low-risk disease and remain favorable even in high-risk metastatic cases when treated promptly with multi-agent chemotherapy [6,7]. Early detection, timely initiation of therapy, and close post-treatment surveillance with serial β-hCG monitoring are critical for achieving remission and detecting recurrence [4,6,7]. Advances in standardized treatment protocols and multidisciplinary oncologic care have substantially improved long-term outcomes.

## Case presentation

A 24-year-old woman presented to the emergency department with persistent vaginal bleeding, progressively worsening pelvic pain, exertional dyspnea, non-bloody non-bilious emesis, and one episode of melena. Her symptoms began approximately two weeks after undergoing dilation and curettage for a molar pregnancy and progressively worsened over the subsequent month. Initially, she experienced intermittent spotting and mild pelvic discomfort. Over time, the bleeding became heavier and continuous, accompanied by increasing fatigue, lightheadedness, and shortness of breath with minimal exertion.

On presentation, vital signs revealed tachycardia (heart rate 122 beats per minute), blood pressure 98/60 mmHg, respiratory rate 22 breaths per minute, oxygen saturation 94% on room air, and temperature 36.8°C. She appeared markedly pale and fatigued. Cardiovascular examination showed sinus tachycardia without murmurs. Pulmonary examination demonstrated mild tachypnea with scattered bilateral crackles. Abdominal examination revealed mild diffuse tenderness without rebound or guarding. Pelvic examination showed active vaginal bleeding without evidence of retained products.

Laboratory evaluation demonstrated a markedly elevated β-hCG level exceeding 250,000 mIU/mL (reference range: 0-5 mIU/mL) and profound anemia with a hemoglobin of 2 g/dL (reference range: 12-15.3 g/dL). Mild transaminitis was noted. Coagulation parameters were within normal limits.

**Figure 1:**
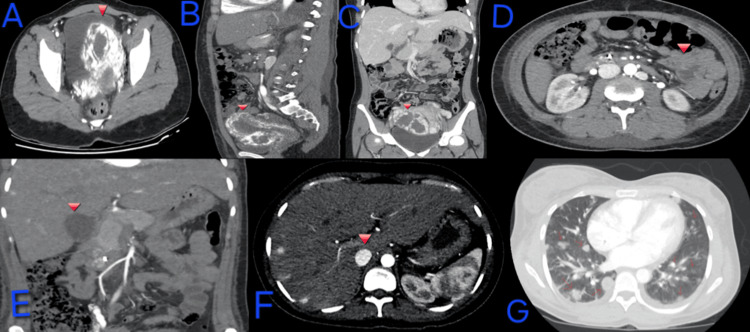
Diagnostic imaging demonstrating uterine primary lesion and multi-organ metastatic disease consistent with high-risk gestational choriocarcinoma. (A-C) Contrast-enhanced CT abdomen/pelvis demonstrating a heterogeneous uterine mass with mixed attenuation and peripheral calcifications, concerning for invasive gestational trophoblastic neoplasia. (D) CT angiography demonstrating an enhancing gastrointestinal lesion consistent with metastatic involvement, correlating with melena. (E-F) Arterially enhancing hepatic lesions consistent with hypervascular metastases. (G) Chest CT demonstrating multiple bilateral pulmonary nodules in a hematogenous “cannonball” distribution, consistent with metastatic spread.

Prior contrast-enhanced CT of the abdomen and pelvis had demonstrated a large heterogeneous uterine mass with mixed attenuation and peripheral calcifications (Figures 1A-1C). Chest CT revealed multiple bilateral mass-like pulmonary opacities.

Pelvic ultrasound showed a 3.9 × 5.2 × 2.5 cm complex heterogeneous lesion centered within the myometrium, with increased internal vascularity and poor delineation of the endometrium, findings concerning for invasive GTN. Hypovascular material within the endometrial cavity was favored to represent blood products rather than retained products of conception.

On current admission, CT angiography of the abdomen and pelvis showed enhancing foci within the gastrointestinal tract (Figure 1D), likely explaining the episode of melena. Additionally, numerous arterially enhancing hepatic lesions consistent with hypervascular metastases, including a representative 1.8 × 1.1 cm lesion (Figures 1E, 1F), were also shown. Repeat chest CT demonstrated innumerable bilateral pulmonary nodules involving all lobes, several exhibiting a classic “cannonball” appearance, with a representative right upper lobe lesion measuring 2.3 × 1.9 cm (Figure 1G).

Given the markedly elevated β-hCG, antecedent molar pregnancy, hypervascular uterine mass, and widespread hematogenous metastases to the lungs, liver, and gastrointestinal tract, the findings were diagnostic of metastatic gestational choriocarcinoma.

Using the FIGO 2000 staging and WHO scoring system, the patient was classified as FIGO Stage IV disease due to distant organ metastases beyond the lungs (liver and gastrointestinal involvement). Her calculated WHO/FIGO prognostic score was ≥12, placing her in the high-risk category (score ≥7). Contributing factors included extremely elevated pretreatment β-hCG levels (>100,000 mIU/mL), liver metastases, multiple metastatic sites, and an interval from antecedent pregnancy of less than four months.

The patient was emergently stabilized with multiple packed red blood cell transfusions and intravenous fluid resuscitation. Given the high-risk classification, she was promptly initiated on multi-agent chemotherapy with etoposide, dactinomycin, methotrexate, cyclophosphamide, and vincristine (EMA/CO), consistent with standard treatment recommendations for high-risk GTN.

**Figure 2:**
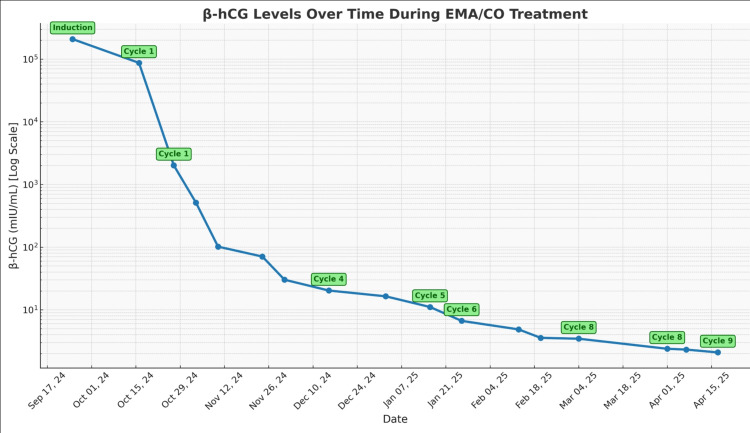
Serial decline in β-hCG levels following induction and EMA/CO chemotherapy in high-risk choriocarcinoma. β-hCG values (mIU/mL; logarithmic scale) are plotted over time with labeled treatment cycles and key clinical milestones. The progressive decline and subsequent normalization of β-hCG demonstrate biochemical remission and correlate with interval radiologic response on follow-up imaging (see Figure 3 and Table 1). EMA/CO: Etoposide, dactinomycin, methotrexate, cyclophosphamide, and vincristine

Serial β-hCG levels demonstrated a progressive decline and eventual normalization following several cycles of therapy, indicating biochemical remission (Figure 2).

**Figure 3:**
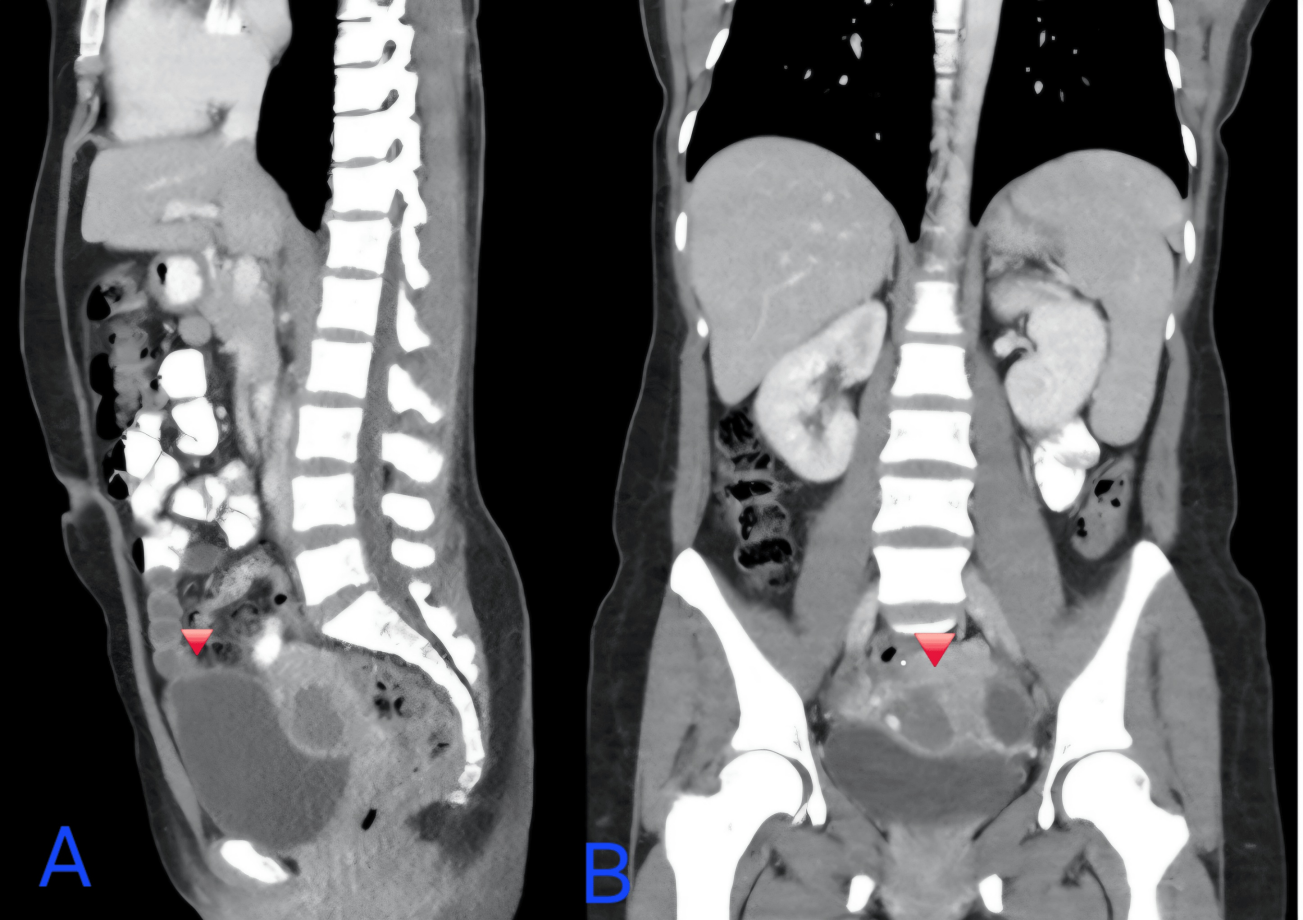
A, B: Previously hypervascular uterine mass, less apparent in this study.

Repeat CT imaging performed seven months later showed complete resolution of hepatic metastases and a significant reduction in the uterine mass, consistent with radiologic response (Figure 3).

In high-risk metastatic choriocarcinoma, remission rates exceed 80-90% with timely multi-agent chemotherapy, even in the presence of extensive metastatic disease. This case underscores the critical importance of early recognition, accurate risk stratification using the FIGO scoring system, and prompt initiation of appropriate therapy. 

To correlate biochemical response with radiologic response, we added a summary table integrating serial β-hCG trends, key imaging findings, and major clinical events (Table 1). This table is intended to provide a clear longitudinal view of disease burden and response to therapy, complementing Figure 1 (extent of metastatic disease at diagnosis) and Figure 2 (β-hCG response over time)

**Table 1:** Correlation of clinical events, laboratory values, and imaging findings. EMA/CO: Etoposide, dactinomycin, methotrexate, cyclophosphamide, and vincristine

Time Point	β-hCG (mIU/mL)	Hemoglobin (g/dL)	Key Imaging Findings	Clinical Events
Post-D&C (Two weeks)	Rising (not quantified)	Not available	Uterine mass noted	Persistent bleeding begins
Admission	>250,000	2	Uterine mass; diffuse pulmonary nodules; liver lesions	Severe anemia; transfusions initiated
After Cycle 2	Significant decline	Stabilizing	No new lesions	Clinical improvement
After completion of EMA/CO	Normalized	Normalized	Resolution of liver metastases; reduced uterine mass	Biochemical remission
Seven-month follow-up	Undetectable	Normal	No active metastatic disease	Radiologic remission

## Discussion

Choriocarcinoma is a potentially highly malignant form of gestational trophoblastic disease (GTD), known for its aggressive behavior and increased chances of metastasis [1]. Although relatively rare, it occurs in approximately 1 in 20,000 to 40,000 pregnancies in the United States [2]. It presents significant clinical challenges due to its ability to develop after varied gestational events, such as molar pregnancy. More specifically, roughly half of all cases follow a normal pregnancy, while molar pregnancies and other gestational events each account for about 25% of clinical courses leading to the diagnosis of choriocarcinoma [2,3]. This highlights the necessity for clinicians to remain vigilant and maintain an increased index of suspicion for choriocarcinoma in all reproductive-age women presenting with abnormal vaginal bleeding, elevated β-hCG levels, or metastatic disease, including those with a prior history of molar pregnancy such as the patient in this case [3]. Metastatic disease involving the liver, gastrointestinal tract and lungs as presented in our patient is a common finding in progressive choriocarcinoma and should be immediately treated as advanced-stage disease. Though choriocarcinoma is more commonly diagnosed in reproductive ages of either extreme, it can and does occur in women of all ages [2].

A crucial component of successfully managing choriocarcinoma is timely diagnosis and early initiation of appropriate therapy. Serial monitoring of serum β-hCG levels post-molar evacuation is paramount, as elevated or rising levels after a dilation and curettage procedure may signal malignant transformation requiring further evaluation and intervention. Literature consistently highlights that delays in diagnosis correlate directly with poorer outcomes, increased morbidity, and mortality due to metastatic disease [1,2]. Thus, adherence to established guidelines recommending close follow-up appointments and prompt intervention based on hCG monitoring is pivotal to patient success [1,3].

In our case, the timely detection and aggressive management of choriocarcinoma following molar pregnancy promoted a favorable outcome. This scenario aligns with existing reports demonstrating strong cure rates when early and appropriate chemotherapy is administered, particularly using methotrexate or multi-agent chemotherapy regimens in higher-risk cases such as ours [4,5]. An EMA/CO multi-agent regimen was successfully used to treat the disease, resulting in clinical improvement and down-trending β-hCG levels to a minimally detectable value, as shown in Figure 2. Current standards of care, including the FIGO criteria, emphasize individual risk stratification to determine optimal therapeutic approaches, reinforcing the importance of personalized medicine in achieving successful outcomes [3,4,6]. 

Risk stratification in GTN is standardized using the FIGO anatomic staging system combined with the WHO/FIGO prognostic scoring system. A score of 0-6 defines low-risk disease, whereas a score ≥7 defines high-risk disease and guides the use of multi-agent chemotherapy regimens [1,2]. Stage IV disease, defined by metastases to organs beyond the lungs and genital tract (e.g., liver, brain), carries a higher mortality risk and requires aggressive multi-agent therapy [2,5].

Current standard management for high-risk GTN consists of EMA/CO (etoposide, methotrexate, dactinomycin, cyclophosphamide, vincristine), which has demonstrated overall survival rates ranging from 80 to 90% even in metastatic disease when treated promptly at specialized centers [3]. Large cohort analyses have reported remission rates exceeding 85% in high-risk patients receiving EMA/CO, though outcomes are less favorable in those with liver or brain metastases and extremely elevated pretreatment β-hCG levels [3,8].

In this case, the patient met criteria for FIGO stage IV high-risk disease due to liver and gastrointestinal metastases and a markedly elevated pretreatment β-hCG (>100,000 mIU/mL). Despite these adverse prognostic features, she demonstrated rapid biochemical remission with normalization of β-hCG and concordant radiologic resolution of metastatic lesions. Her favorable response aligns with reported outcomes demonstrating that choriocarcinoma remains highly chemosensitive when managed with risk-adapted multi-agent therapy and close surveillance [3,8].

## Conclusions

In conclusion, while diagnosing choriocarcinoma can be complex, a thorough gynecological history, elevated β-hCG levels, and characteristic ultrasound findings are typically sufficient to establish the diagnosis. Ultimately, this case reinforces the continued need for education and awareness among healthcare providers regarding the risk factors, diagnostic strategies, and treatments associated with choriocarcinoma. Early identification through surveillance and the prompt initiation of treatment significantly reduce morbidity and improve prognosis. This case underscores the vital role of ongoing clinical vigilance in managing GTD and its malignant transformation. With prompt diagnosis and management in a specialized center, the prognosis is excellent. Additionally, recognizing evolving patterns of presentation and ensuring patient compliance are essential in reducing complications and overall treatment costs.
